# Is Dentistry a Charitable Profession?

**Published:** 1900-05

**Authors:** 


### Editorial.

### IS DENTISTRY A CHARITABLE PROFESSION?

The question of charitable work in dentistry was introduced at a recent meeting of The New York Institute of Stomatology by a paper presented by Dr. A. J. Flanagan.

The discussion of this essay brought out the views not only of the dentists present, but also those of several prominent visiting members of the medical profession. The full report appeared in the April number of this journal.

The question is one by no means new, for it is as old as dentistry, but it has probably not been presented before in a concrete form for discussion and general consideration.

It is proposed in New York to establish a dental dispensary for the treatment of the poor, and some of the members present were willing to devote a half-day in each week gratuitously to this service. The adoption of this plan is not an original effort, for it was tried and failed in Brooklyn some years since.

Medical men have repeatedly stated that dentistry could not be called a profession for one important reason,—that it did not give its services free to the poor; and this charge was repeated at this meeting. It was also charged that dentists would not give their time to the dispensaries unless paid for the service. In fact, it was assumed that dentistry had no dispensaries, and did practically nothing to relieve the crying needs of the great undercurrent life of our large cities. It was further broadly stated that the work performed at the colleges stood in no near relation to that of the dispensaries connected with hospitals, or those working independent of them.

The relation which dentistry holds to the general public is not well understood by medical men. They seem incapable of comprehending the wide distinction to be drawn between dental and medical practice, and, therefore, when they assume to define a profession as a body of men serving the poor gratuitously, and that dentistry, failing in this, is therefore not a profession, they simply fail to comprehend the true character of dental work and its limitations.

Is it true that dentists do nothing in the way of professional charity? To answer this some allusion must be made to the beginning of dentistry as a profession. This must be dated from the first organization of a dental college by Dr. Chapin A. Harris in 1839. It was clearly evident to this broad-minded man that a college without a dispensary attached would be a failure. His idea extended beyond that of the medical dispensary in that he regarded the combination of free service and practice as part of the educational training. This was not new, for it was similarly regarded in medical dispensaries, but with this marked difference, the medical dispensary was given over to a few favored graduates, while the majority of medical students were deprived of all practical experience. On the other hand, the dental dispensary was made the means of increasing practical skill and fitting thereby the undergraduate with an experience upon the living subject enabling him at once, on entering the larger arena of practice, to fulfil all the duties of his profession. The result of this initial experiment was so satisfactory that there is not a dental college in the world to-day that does not regard its dispensary as the most important part of its training. Indeed, without it there is not a dental college that could possibly be sustained. Hence the "clinic," as it is generally called, is jealously guarded and every effort made to encourage the poor to attend and receive careful attention under the care of competent instructors.

It will doubtless be said, indeed was intimated in the discussion, that this was not the charity desired. In fact, it was not charity at all, and could not be classed with medical dispensaries, where treatment was given without charge to those absolutely unable to pay for medical service. This may be true, but if so, why the denunciations in medical journals of these same dispensaries that they do not confine their work to the real poor, but that large numbers able to pay are recipients of the service; in fact, that no discrimination is made, thus taking the bread from the mouths of young practitioners. The same cry has been heard over and over again in regard to dental clinics, and it is no doubt true that sufficient care is not observed in both of these departments of practice.

Will the establishment of the dental dispensary, separate from college work, overcome this difficulty? The answer must be decidedly in the negative. Human nature is the same everywhere. To get something for nothing is a constant temptation, and the expedients resorted to to accomplish this would, if told, be an interesting history both in medical and dental experience.

Is it possible to work for the very poor in dentistry? This is another very large question. When the practice of dentistry is compared with that of medicine, we are met at the threshold of our inquiry with the fact that methods of treatment stand in no harmonious relation. The medical practitioner can make a hasty examination of this patient, write a prescription, occupying perhaps five or ten minutes of time; another patient is then taken. The work of the surgeon comes more nearly that of the dentist. The specialists, as the ophthalmologist, aurist, laryngologist, etc., occupy also very close relations, but even these, in point of time consumed, or in exhaustive labor, cannot compare with dentistry, nor is the work as disagreeable in some of its aspects. Now, what has the dentist to meet? He must first have a complicated set of instruments. These must be supplied, in part at least, by those in charge. He must have each patient under his care for at least an hour, possibly longer. He is brought necessarily in very close contact throughout that period, and must suffer all the disagreeableness of this close communion with the great unwashed; and then, to crown all, he knows that his effort will be entirely nugatory through the wretched environment of these people and the utter neglect of all hygienic conditions in the oral cavity. Can any one think of treating gingivitis, pyorrhœa alveolaris, and the host of pathological conditions in mouths in which the tooth-brush is an entire stranger? All know that such treatment could have no good result. It is a dream of the philanthropist, to be dissipated by the stern realities of fact. Admitting that these obstructions to good practice could be overcome, how could this class give the time necessary for extended dental operations? The question carries with it its own answer: it is impossible. Until the slums have ceased to exist, and men and women are raised to a higher level, the forceps will continue to be the instrument of relief. There is not a right-thinking person but would wish it otherwise, but the stern logic of facts makes this impossible in our present imperfect civilization.

There is another class, very limited in means, that can be benefited, and it is to this body of men and women that true charity may be extended. This may be represented by girls engaged in various avocations, young men of limited means supporting a family on a meagre income, children in our public institutions, institutions for the insane, etc. Provision is made in all our large cities for a portion of this class in institutions, but the provision is entirely inadequate to the demand for dental service. In smaller towns this problem remains unsolved, and seemingly no attempt is being made to effect a solution either in medicine or dentistry.

This brings the subject to the consideration of the college clinic. Does it bear a true relation to the medical dispensary, and is it in any sense a charitable institution? It is somewhat difficult to answer this question, for this would require a very extended knowledge of the practice in vogue in various dental colleges. It is assumed, however, that this is very much the same in all, and the endeavor, universally, is to bring all operations to nearly absolute cost. One college known to the writer makes no charge whatever for anything but operations with gold in the dispensary, and absolute cost for all other operations. All dental dispensaries could not do this, as the great expense would constitute a severe strain upon the resources of the institutions, hence a small fee in some is demanded. The operators in all cases are the students, with occasional personal assistance in difficult cases. In oral surgery all operations are, of course, free, and these are performed by the professor in charge.

It is impossible to conceive of any dispensary on an independent foundation that could do more than this. If it were started on an entirely free basis, its life would necessarily be very brief. Somebody must continually pay the bills, as well as give the time, two things that speedily end in the conviction that "true charity begins at home." If some philanthropist could be found to endow an institution of this kind, it is feared it would eventually degenerate into a poorly conducted school for undergraduates.

When medical men assume that the dental profession fails in its duty in not attending to the wants of the poor, or those of limited means, they talk in ignorance. It is safe to presume that these have never entered the dispensary of a well-equipped dental college during the busy hours of the day. The institution with which the writer is most familiar has one hundred chairs in one department, usually all filled with patients waiting. The last report of this college gave a record of 7235 patients and 18,638 operations in the operative dispensary, of which, 13,678 were absolutely free. This amount of charitable work could hardly be equalled by any private dental dispensary, however well conducted; and when the amount of labor involved is taken into consideration, it could probably not be approached by any medical institution. It must further be considered that in the same city there are three other dental schools all doing proportionate good work in the same direction. The criticism will probably be made that all this is not true charity, as it lacks the altruistic motive. While it is true there is a selfish side to it, the same plan exists in medical dispensaries. They are not established for the entire good of the patients. It is a question whether self is ever entirely eliminated from benevolent deeds. The answer to all such criticism must be found in the result that thousands are helped and, what is of equal importance, are being instructed in the care of the oral cavity, and thereby helping to prevent not only disease there, but throughout the entire organism.

While the writer is in full sympathy with the general trend of thought in that discussion, it does seem that with our charitable views we should combine the practical possibilities, and these would appear to be confined to the colleges that are alone capable of bringing together the needy, willing to suffer, and the undergraduate willing and eager to work.

